# Current and future options for treating complicated skin and soft tissue infections: focus on fluoroquinolones and long-acting lipoglycopeptide antibiotics

**DOI:** 10.1093/jac/dkab351

**Published:** 2021-11-21

**Authors:** Christian Eckmann, Paul M Tulkens

**Affiliations:** 1 Department of General, Visceral and Thoracic Surgery, Klinikum Hannoversch-Muenden, Goettingen University, Germany; 2 Cellular and Molecular Pharmacology, Louvain Drug Research Institute, Université catholique de Louvain (UCLouvain), Brussels, Belgium

## Abstract

Bacterial skin and soft tissue infections are among the most common bacterial infections and constitute a major burden for patients and healthcare systems. Care is complicated by the variety of potential pathogens, some with resistance to previously effective antimicrobial agents, the wide spectrum of clinical presentations and the risk of progression to life-threatening forms. More-efficient care pathways are needed that can reduce hospital admissions and length of stay, while maintaining a high quality of care and adhering to antimicrobial stewardship principles. Several agents approved recently for treating acute bacterial skin and skin structure infections have characteristics that meet these requirements. We address the clinical and pharmacological characteristics of the fourth-generation fluoroquinolone delafloxacin, and the long-acting lipoglycopeptide agents dalbavancin and oritavancin.

## Introduction

Infections of the skin and subcutaneous tissue, muscle and fascia are commonly referred to as skin and soft-tissue infections (SSTIs) and are frequent reasons for medical visits in both inpatient and outpatient settings worldwide,^1–4^ They include a wide variety of conditions that range from simple superficial infections to complicated surgical wound infections and rapidly progressing necrotizing fasciitis.^4^^,^^5^ The most common types are cellulitis and abscesses.^3^^,^^6^ A subset of severe SSTIs referred to in the literature as complicated SSTIs (cSSTIs) or complicated skin and skin structure infections (cSSSI) require early prompt treatment that generally includes both surgery with drainage and debridement, and appropriate antibiotic therapy.^7^^,^^8^ cSSTIs may be further classified as necrotizing or non-necrotizing.^8^^,^^9^ Common cSSTIs include surgical site or traumatic wound infections, diabetic foot infections, perianal abscesses, and extensive cellulitis, or infections occurring in patients with significant comorbidities that affect the therapeutic response.^10^^,^^11^ These infections represent a substantial healthcare burden.^12^

In 2013 the US FDA defined acute bacterial skin and skin structure infections (ABSSSI) as the subset of cSSTIs that includes cellulitis/erysipelas, wound infections, and major cutaneous abscess, while excluding deeper infections of muscle and fascia, necrotizing infections and diabetic foot infections.^13^ The definition of ABSSSI imposes a minimum lesion surface area of ≥75 cm^2^, and the primary response criteria are defined as a ≥ 20% reduction in lesion size with resolution of fever after 48–72 h in patients who are alive and did not receive rescue therapy. For clinical practice, this definition may provide a framework for assessing the effectiveness of treatment in terms of early response,^14^^,^^15^ although early response can be influenced by factors other than antimicrobial treatment,^16^ and the duration of response must be confirmed.

EMA guidelines on the evaluation of medicinal products for treatment of bacterial infections indicate that registration studies in ABSSSI may enrol patients with cellulitis, erysipelas, traumatic or post-surgical wound infections, and major abscesses; whereas, patients with confirmed or suspected osteomyelitis, septic arthritis or severe necrotizing infections should be excluded.^17^ A minimum infection area or estimated abscess dimensions should be declared in the study protocol, and the proportion of patients with abscesses should be limited to about 30%. Drainage, if necessary, should be conducted around the time of randomization. The primary analysis should be the clinical outcome in the ITT population, using a non-inferiority margin of 10%, assessed at the test-of-cure visit, the timing of which is based on the maximum treatment duration and the elimination half-lives of test and comparative agents.^17^

## Bacterial ecology

The choice of empirical antimicrobial treatment for SSTIs can be challenging due to the geographically varied and changing local epidemiology of community-acquired and healthcare-acquired infections. Treatment choices should be informed by local surveillance data. In general, Gram-positive organisms are involved most frequently, with *Staphylococcus aureus,* β-haemolytic streptococci, *Enterococcus* spp., and coagulase-negative staphylococci representing the most common pathogens identified.^11^^,^^18^ Gram-negative pathogens, especially *Escherichia coli*, may be more common in healthcare-associated cSSTIs than in community-associated cSSTIs,^19^ and are often associated with surgical site infections (SSIs) after abdominal surgery and perineal abscesses.^11^^,^^20^

In general, most ABSSSIs are caused by *Staphylococcus aureus*, including MRSA, and *Streptococcus pyogenes*; less-common causes include other *Streptococcus* species, and Gram-negative bacteria.^13^^,^^18^

### MRSA global prevalence

In many parts of the world, treatment of infections is complicated by antimicrobial-resistant pathogens. MRSA is one of the most important examples. Its prevalence varies by geographic region and changes over time. In Europe, the involvement of MRSA among surveyed infections varies widely by country (mean 16.4%, range 0%–43.0%) and has declined in many countries in 2018.^21^ MRSA was the second most frequent resistant pathogen responsible for cases and attributable deaths in the European Union.^22^ In some geographic regions, community-associated MRSA has emerged as a major threat,^23^ as virulent clones arise and are disseminated,^24^^,^^25^ a phenomenon accelerated by international travel.^26^

Considering specifically skin infections, a global survey of community-acquired ABSSSI identified MRSA in 18.5% of cultured pathogens, with substantial variability according to geographic region, ranging from 15.8% in Eastern Europe and the Mediterranean region to 21.4% in the Asia-Pacific region.^18^ In the US, a retrospective population-based study of patients with SSTIs revealed that 46% of cultured *S. aureus* were MRSA.^20^ The emergence of MRSA has changed antimicrobial prescribing for skin infections.^27^

### Recurrence and relapses

Staphylococcal infections show a tendency for recurrence despite well-conducted treatment with antibiotics considered as suitable based on susceptibility testing. This has been attributed to colonization,^28^ and may also be associated with the capacity of staphylococci to survive intracellularly,^29^^,^^30^ which has been suggested to play a key role in treatment failure in chronic and relapsing staphylococcal infections.^31^ Recently, persistent intracellular presence has been implicated as an explanation for the relapse/recurrence of *S. aureus* infections.^32^

### Consequences of under- and over-treatment

An inappropriate level of care for patients with cSSTI can expose the patient to avoidable risk and lead to unnecessary use of health resources. Undertreatment of serious infections is a common problem.^33^^,^^34^ This occurs when an inappropriate empirical antimicrobial regimen does not cover the causative pathogen.^35–38^ cSSTIs involving MRSA, Gram-negative pathogens or polymicrobial infections are associated with a higher risk of inappropriate empirical treatment.^37^^,^^38^ Some MRSA strains are also resistant to other frequently used agents, including macrolides, fluoroquinolones and linezolid.^39^^,^^40^ Undertreatment, even with active antibiotics, may contribute to the maintenance of persistent extracellular and intracellular bacteria leading to recurrent infection. A large observational study conducted in 10 European countries revealed that inappropriate initial treatment of cSSTI resulted in an approximate doubling of the length of stay (LOS).^41^

Overtreatment of uncomplicated SSTIs (e.g. non-guideline-concordant antibiotic prescription) is also a common problem.^42–46^ This results in avoidable exposure to the risk of drug-related adverse events, unnecessary costs for healthcare systems and is at odds with principles of antimicrobial stewardship. Current guidelines recommend that erysipelas, impetigo, folliculitis and simple abscesses may be treated with antibiotics or drainage alone in immunocompetent patients without signs of systemic inflammatory response.^8^^,^^9^ See the latest guidelines for indications for broad-spectrum antibiotics.^8^

### Available treatments

Traditional oral antimicrobial agents for SSTIs have included: (i) a series of old but still effective antibiotics (depending on local trends of resistance) such as clindamycin, doxycycline, or trimethoprim/sulfamethoxazole, all of which can be given orally; (ii) β-lactamase-resistant penicillins and cephalosporins (considered as first choice for infections caused by methicillin-susceptible staphylococci); and (iii) vancomycin or linezolid (especially in a context of methicillin-resistant organisms). More recent additions have included daptomycin (a lipopeptide), tedizolid (an oxazolidinone), and the anti-MRSA cephalosporins (ceftaroline and, in some markets, ceftobiprole). All these agents, with the notable exception of linezolid and tedizolid, are strictly intravenous drugs; daptomycin and tedizolid are administered once daily. Toxicity issues with older drugs include bone marrow toxicity for linezolid and nephrotoxicity for vancomycin and possibly for vancomycin plus piperacillin/tazobactam.^12^^,^^47^

The emergence of multidrug-resistant MRSA and VRE, combined with the need for extended-spectrum agents that are better tolerated and more convenient to use, has led to the development of new long-acting intravenous agents such as dalbavancin and oritavancin that can be given once a week or even once only. Novel agents with oral and IV formulations such as delafloxacin are also of obvious interest. Effective agents for treating cSSTIs must cover the likely causative pathogens and have high skin and soft tissue penetration. The choice must also consider patient comorbidities, potential side effects, and economic issues.

### Economic issues

**Figure 1.**
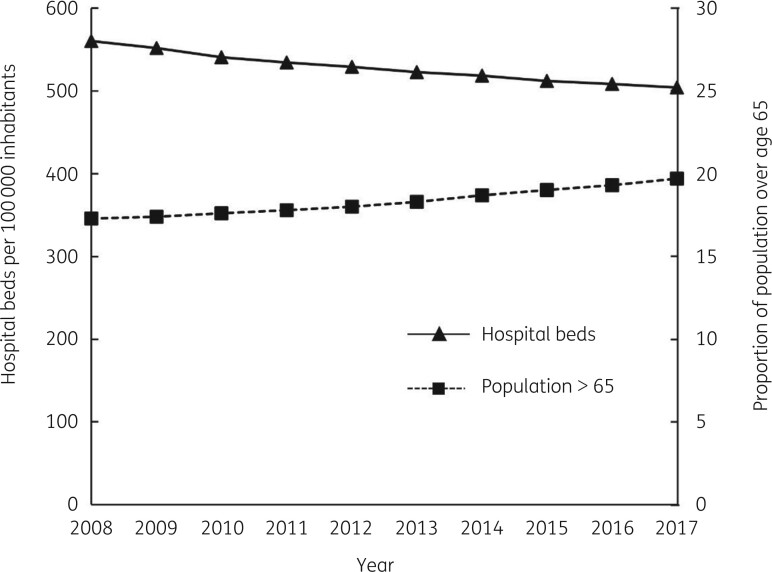
Trends in European hospital capacity (beds per 100 000 inhabitants) and the proportion of the population age >65 years. Figure constructed using data from Eurostat.^48^^,^^49^

Healthcare providers in most countries are under pressure to improve cost-effectiveness. In Europe, there has been a long-term trend toward reduction in hospital capacity, while the population is also ageing (Figure 1).^48^^,^^49^

**Figure 2.**
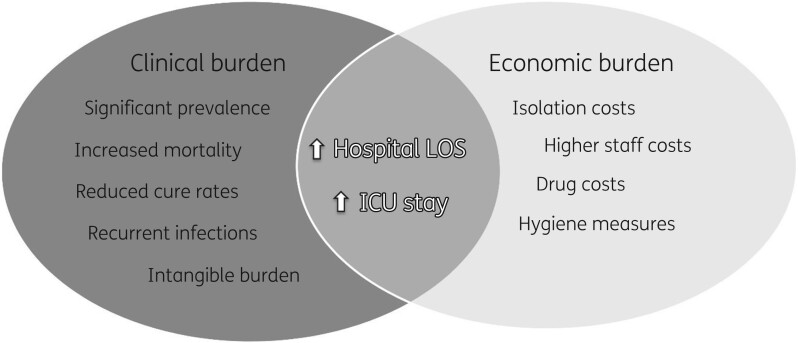
Summary of the clinical and economic burden of MRSA.

The incidence of SSTIs was found to be higher in the elderly population;^20^^,^^50^^,^^51^ moreover, several comorbidities that are more common in the elderly, such as diabetes, liver and kidney disease and vascular insufficiency, also increase the risk of developing complicated/severe SSTI.^52^ Therefore, the current situation of an ageing population with rising SSTI incidence is not compatible with a reduction in hospital capacity. The economic burden of SSTI care in the US is nearly $14 billion per year.^53^ MRSA infections are associated with higher clinical and economic burdens (Figure 2).

Strategies are needed to improve efficiency by avoiding unnecessary admissions or by reducing LOS. Outpatient administration of antimicrobial therapy in patients who do not require admission or are eligible for early discharge (ED) is an effective strategy for transferring care to the outpatient setting.^54^ This has the additional advantage of reducing the risk of healthcare-associated infections (HCAIs), which cause considerable additional healthcare burden.^55^

Where available, outpatient parenteral antibiotic therapy (OPAT) programmes are effective for appropriate patients with skin infections.^56–58^ They, however, do require additional resources for organizing and managing the service.^59^^,^^60^ Programmes that promote sequential treatment with IV and oral antimicrobials (early switch, ES) represent an alternative strategy when the appropriate antimicrobials are available also in oral formulations with good bioavailability and penetration.^61^^,^^62^ Finally, long-acting antimicrobials that provide reliable coverage with a single or weekly IV administration offer an option that may reduce LOS, while overcoming concerns regarding adherence and the need to maintain IV access.^63^

## Delafloxacin

**Figure 3.**
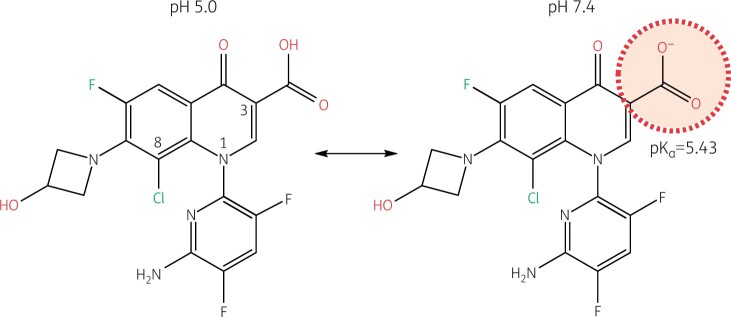
Delafloxacin. The only ionizable group in biological media is the carboxylate carried in position C3 and with a pK_a_ of 5.43 (the hydroxyazetidine group at position 7 is not ionizable in biological media). This causes delafloxacin to be predominantly negatively charged (mono cationic) with an almost equilibrated lipophilic/hydrophilic balance (logD_pH7.4_* = *0.59) in the extracellular milieu (pH 7.4), while becoming predominantly un-ionized and more lipophilic [logD_pH5_ = 2.22 (close to its logP of 2.56)] at pH 5.5 (as can be observed in infected tissues and in intracellular compartments harbouring *S. aureus*). This causes increased accumulation, contributing to increased potency (lower MICs) of delafloxacin in these compartments.^66^^,^^67^ The other important features of delafloxacin include a chlorine substituent in position C8 which enhances activity, and a bulky heteroaromatic group in N1 which increases the drug contact surface.^68^^,^^69^ Structures and calculations were made with Marvin sketch 21.13 (Chemaxon, Budapest, Hungary).

Delafloxacin is a fourth-generation fluoroquinolone with broad-spectrum coverage of Gram-positive and Gram-negative bacteria.^64^^,^^65^ It is the only approved anionic fluoroquinolone (Figure 3).^66–69^

This differentiates it from most other widely used fluroquinolones such as moxifloxacin, levofloxacin, and ciprofloxacin, which are zwitterionic. Together with specific structural determinants (such as a heteroaromatic substituent at position 1 and a chlorine at position 8), the anionic character of delafloxacin markedly contributes to its very low MICs against Gram-positive organisms, such as *S. aureus*, because it allows for much greater uptake by these bacteria under acidic conditions,^67^ while an opposite trend is observed for the zwitterionic agent moxifloxacin.^66^

Once inside bacteria, fluroquinolones bind to both DNA topoisomerase IV and DNA gyrase and inhibit their activities, which are both essential for replication. Fluroquinolones that bind with higher affinity to DNA topoisomerase are more active against Gram-positive bacteria, while fluroquinolones that bind with higher affinity to DNA gyrase are more active against Gram-negative bacteria.^70^ Delafloxacin binds with similar affinity to both targets, which may explain its activity against both Gram-positive and Gram-negative bacteria and is believed to result in decreased emergence of resistance because two simultaneous mutations are required. *In vitro* experiments by Remy *et al.*^71^ showed indeed that, in addition to lower MICs, delafloxacin has a lower mutation prevention concentration/MIC ratio and a higher fitness cost compared with levofloxacin or moxifloxacin.

Delafloxacin has 59% oral bioavailability and a steady-state volume of distribution of 30–48 L,^72^ that combined with its enhanced activity in acidic environments,^67^ make it appropriate for skin infections. It is available in both intravenous and oral formulations, with doses of 300 mg for IV and of 450 mg for oral formulations that are bioequivalent, which facilitates parenteral to oral switching.

Delafloxacin was approved for ABSSSI by the US FDA in 2017 and EMA in 2019.^72^^,^^73^ Approval was based on the results of two similarly designed Phase III randomized, double-blind, studies that compared delafloxacin with the active control vancomycin plus aztreonam in a total of 1510 patients with ABSSSI stratified by infection type. The studies differed primarily in how delafloxacin was administered: in Study 302,^74^ delafloxacin was administered at 300 mg IV every 12 h for 5–14 days; in Study 303,^75^ delafloxacin was administered in an IV to oral switch regimen consisting of delafloxacin 300 mg IV every 12 h for the first 3 days, followed by the bioequivalent oral dose of 450 mg every 12 h for the remainder of the treatment period. The studies differed also in enrolment criteria and analysis of data from obese patients: in Study 302, body weight was limited to 140 kg and patients were not stratified for obesity at enrolment; in Study 303 body weight was limited to 200 kg, obese patients (BMI ≥30 kg/m^2^) were limited to ≤50% of the population, and patients were stratified by BMI above or below 30 kg/m^2^ The primary efficacy endpoint (for the FDA)^13^ in both studies was a ≥ 20% reduction in lesion size at 48 to 72 h in the absence of clinical failure in the ITT population. Response after 14 days and 21–28 days of follow-up were secondary endpoints (primary efficacy endpoint for the EMA).^17^

Study 302 randomized 660 patients to IV delafloxacin (*n = *331) or vancomycin plus aztreonam (*n = *329). *S. aureus* was the most frequently isolated pathogen (66%), of which more than half (52%) were MRSA. Delafloxacin was non-inferior to the active comparator for all endpoints and subgroups. Subsequently, Study 303 randomized 850 patients to the delafloxacin IV to oral switch regimen (*n = *423) or vancomycin plus aztreonam (*n = *427). *S. aureus* was again the most frequently isolated pathogen (58%), of which 36% were MRSA. Sequential IV to oral delafloxacin was non-inferior to IV vancomycin plus aztreonam for all endpoints and subgroups.

An integrated analysis of efficacy data from these two Phase III ABSSSI studies was conducted.^76^ The aggregate study population had considerable characteristics that were evenly distributed between the study arms and included vascular disease (29%), diabetes (11%), obesity (BMI ≥30 m^2^, 42%); 13% of patients were >65 years old. Analysis of aggregated efficacy data from both studies revealed similar objective response rates with delafloxacin and vancomycin/aztreonam after 48–72 h (81.3% versus 80.7%), and investigator-assessed response rates at 14 days (84.7% versus 84.1%) and 21–28 days (82.0% versus 81.7%).^76^ In a study by McCurdy *et al*.,^64^ microbiological response rates among delafloxacin-treated patients were 98.4% for all *S. aureus* isolates (245/249), 98.6% for MRSA isolates (70/71), 98.8% for levofloxacin-non-susceptible *S. aureus* isolates (80/81), and 98.8% for *S. aureus* isolates with documented mutations in the quinolone resistance-determining region (81/82).^64^ Of note, Pfaller *et al.*^65^ showed that in 2014 only 91.2% and 95.3% of the US and EU MRSA isolates, respectively, had an MIC that would qualify them as non-resistant based on EUCAST breakpoints (R > 0.25 mg/L). In a more recent surveillance study, 92.4% of 4484 European *S. aureus* isolates from ABSSSI collected from 2014–19 were susceptible to delafloxacin.^77^ Delafloxacin was more active than levofloxacin (84.0% susceptible with increased exposure) and moxifloxacin (84.3% susceptible). A 2017 surveillance study on 757 *S. aureus* isolates from patients in seven Brooklyn, NY hospitals identified a similar susceptibility rate for *S. aureus* (91%, 689/757), with 16 organisms having an MIC ≥2 mg/L, i.e. at or above the FDA resistant breakpoint (≥1 mg/L) however, only 78% of the MRSA isolates (219/281) were susceptible to delafloxacin using a MIC_50_ value of 0.12 mg/L.^78^ Molecular typing of 16 delafloxacin-resistant MRSA isolates revealed that most were healthcare-associated strains, and sequencing of 6 isolates revealed mutations involving gyrase and topoisomerase IV genes.^78^

### Safety

The most frequent adverse events (AEs) associated with fluoroquinolones are gastrointestinal symptoms, headaches, and altered liver function tests.^79^ Rare AEs of special interest with fluoroquinolones include tendinitis and tendon ruptures, peripheral neuropathy, CNS effects, worsening of myasthenia gravis symptoms, serum glucose disturbances, and development of *Clostridioides difficile*-associated diarrhoea.^80^ In 2018, the FDA added a warning about potential aortic aneurysm/dissections.^81^ A pooled analysis (*n = *1510) of adverse events in the delafloxacin Phase III studies 302 and 303 revealed similar rates of treatment-emergent AEs (45.1% and 47.7%) and treatment-related AEs (22.1% and 26.1%) in the delafloxacin and comparator (vancomycin ± aztreonam) groups, respectively.^82^ Of interest, there were fewer treatment-emergent AEs of special interest with fluoroquinolones in the delafloxacin arm (7.0%) than in the comparator arm (9.2%). No discontinuation in the delafloxacin group was due to fluroquinolone-related AEs.^82^ This was confirmed in a Phase III study of community-acquired bacterial pneumonia in an older population with more comorbidities, which randomized 431 patients to delafloxacin versus moxifloxacin and reported AEs of special interest with fluoroquinolones in 7.9% (34/429) of patients in the delafloxacin arm and 7.5% (32/427) in the moxifloxacin arm.^83^ In healthy volunteers, delafloxacin was not associated with the delayed ventricular repolarization and resulting elongation of the electrocardiogram QT-segment that characterizes other agents in the class,^84^ which may be important when treating patients at risk for arrhythmias. Although delafloxacin was well tolerated in these studies, as well as in early phase studies,^80^ these encouraging results need to be confirmed by pharmacovigilance data able to detect these rare events. Meanwhile, fluoroquinolone use is reserved for severe infections and patients who do not have other treatment options.

Delafloxacin has several characteristics that may make it an appropriate choice for the treatment of skin infections. First, its bioavailability and pharmacokinetic profile, with a 10 h half-life that allows twice-daily administration, its primarily renal elimination, weak interactions with liver cytochrome P-450 drug-metabolizing enzymes, glucuronyltransferases, and drug transporters make its use possible in most patients, with no dose adaptation needed in case of liver insufficiency and dose reduction only necessary in patients with severe renal impairment (creatinine clearance <30 mL/min) who are receiving the IV formulation. These characteristics allow safe and effective sequential IV to oral therapy in most patients and may greatly facilitate the early discharge of patients with ABSSSI and support antibiotic stewardship strategies with significant benefits for healthcare resource utilization and clinical outcomes.

Second, delafloxacin clinical breakpoints for Gram-positive organisms are above the MIC_90_ for most clinical isolates (Table 1).

**Table 1.** Breakpoints (mg/L) of delafloxacin for organisms causing acute bacterial skin and skin structure infections (ABSSSI)

Pathogen	FDA^a^			EUCAST (EMA)^b^	
	S^c^	I	R	S	R
*Staphylococcus aureus* ^d^	≤0.25	0.5	≥1	≤0.25	>0.25
*Staphylococcus haemolyticus*	≤0.25	0.5	≥1		
*Staphylococcus lugdunensis*	≤0.03	^e^	^e^		
*Streptococcus* groups A, B, C and G				≤0.03	>0.03
*Streptococcus pyogenes*	≤0.03	^e^	^e^		
*Streptococcus agalactiae*	≤0.06	0.12	≥0.25		
*Streptococcus anginosus* group^f^	≤0.06	^e^	^e^	≤0.03	>0.03
*Enterococcus* spp.				IE^g^	IE
*Enterococcus faecalis*	≤0.12	0.25	≥0.5		
Viridans group				≤0.03	>0.03
Enterobacterales^h^^,^^i^	≤0.25	0.5	≥1	≤0.125	>0.125
*Pseudomonas aeruginosa*	≤0.5	1	≥2	IE	IE

a US FDA Recognized Antimicrobial Susceptibility Test Interpretive Criteria.

b EUCAST [acting under Standard Operating Procedures (SOP) for the EMA].

c S, susceptible; I, intermediate; R, resistant [FDA and EUCAST; see detailed definition in (i) Document M100 (Performance Standards for Antimicrobial Susceptibility Testing) of the CLSI for the FDA and (ii) EUCAST documentation (EUCAST; no I category set for delafloxacin essentially because the approved dosage is fixed)].

d Methicillin-resistant and methicillin-susceptible isolates.

e For the FDA, the current absence of resistant isolates precludes defining any results other than ‘Susceptible’. Isolates yielding MIC results other than ‘Susceptible’ should be submitted to a reference laboratory for further testing.

f For FDA: this includes *S. anginosus, S. constellatus and S. intermedius*.

g IE (EUCAST): insufficient evidence that the organism or group is a good target for therapy with the agent.

h New nomenclature for Enterobacteriaceae.

i For FDA: *E. coli, K. pneumoniae*, and *E. cloacae* only; for EUCAST: *E coli* only.

Third, unlike several other antibiotics narrowly oriented towards Gram-positive organisms, delafloxacin shows useful activity against a limited but significant proportion of important Gram-negative pathogens. Thus, delafloxacin is approved by both the FDA and the EMA for ABSSSI caused by *E. coli, Klebsiella pneumoniae, Enterobacter cloacae*, and *Pseudomonas aeruginosa*, plus, for the EMA, *Klebsiella oxytoca and Proteus mirabilis*. However, susceptibility testing of Gram-negative organisms is probably essential at both the population and individual levels, since the MIC_90_ of several of them may exceed the resistance breakpoints [≥1 mg/L for Enterobacterales and *P. aeruginosa* for the FDA, and >0.125 mg/L for EUCAST (Enterobacterales only; EUCAST found insufficient evidence (IE) for setting breakpoints for *P. aeruginosa* and the other Gram-negative organisms included in the list of infections that could be treated with delafloxacin)].

Fourth, delafloxacin is effective against *S. aureus* from biofilms, under conditions where many other antibiotics failed,^85^ probably because of its enhanced activity at acid pH as discussed above, combined with its bactericidal activity against intracellular bacteria.^85^

## Long-acting lipoglycopeptides

### Basic data and assessment of value

Long-acting lipoglycopeptide antimicrobials represent another strategy for achieving ED. Their long half-lives allow treatment of ABSSSI with a single or weekly IV dose, providing long-term IV treatment without requiring continuous IV access or inpatient stay. Lipoglycopeptides are very large molecules compared with most antibiotics. This prevents them from crossing the outer bacterial membrane and cell wall of Gram-negative organisms, strictly limiting their spectrum of activity to Gram-positive bacteria. It also explains their complete lack of bioavailability through the intestinal barrier and requirement for parenteral administration.

**Figure 4.**
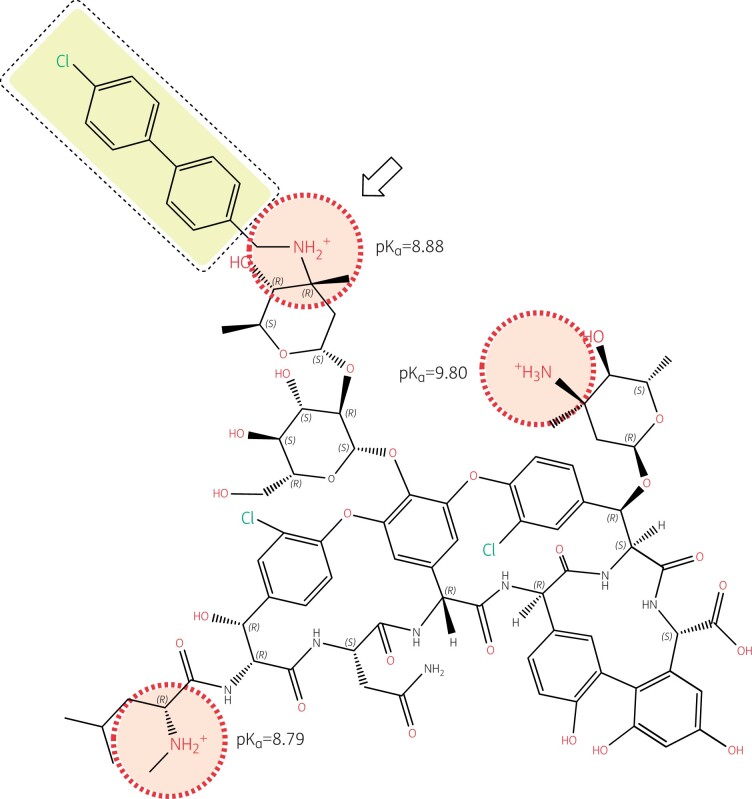
Structure of oritavancin with emphasis on its ionizable functions and hydrophobic side chain. Oritavancin is a large molecule compared to most other antibiotics (projection area: 183–281 Å^2^; van der Waals volume: 1254 Å^3^), which explains its lack of penetration through the outer membrane of Gram-negative organisms, hence its lack of activity against these pathogens, as well as its complete lack of oral bioavailability and necessity for parenteral administration. While the un-ionized form of oritavancin is highly lipophilic (logP = 9.32) due to the presence of the 4′-chlorobiphenyl methyl moiety (highlighted in yellow), the molecule is made polycationic at both pH 7.4 and 5.5 due to the presence of 3 amino functions with pK_a_s >8.5, and further gains in polarity due to the large number of ketones and hydroxyl functions with electronic de-localizations (several of which are involved in the binding of the d-Ala-d-Ala termini of the peptidoglycan precursors), showing a globally strong negative logD at both neutral and acidic pHs (−16.67 and −7.33 at pH 7.4 and 5.5, respectively). Oritavancin has, therefore, a marked amphiphilic character favouring its interaction with negatively charged membrane phospholipids (abundant in Gram-positive bacteria). This causes membrane rigidification and permeabilization,^88^^,^^89^ which may contribute to, and enhance the bactericidal activity of oritavancin, including against intracellular,^90^ and biofilm-encased *S. aureus*.^91^^,^^92^ The 4′ chlorobiphenyl methyl increases the binding of oritavancin to the d-Ala-d-Ala terminus of the cell wall precursor so that modification of the terminal d-Ala to d-Lac in vancomycin-resistant organisms using the VanA mechanism is insufficient to markedly affect oritavancin activity.^93^ The correct positioning of the 4′ chlorobiphenyl methyl moiety, however, requires the presence of a closely apposed positive charge (see arrow), which is specific to oritavancin. Structures and calculations were made with Marvin sketch 21.13 (Chemaxon, Budapest, Hungary).

**Figure 5.**
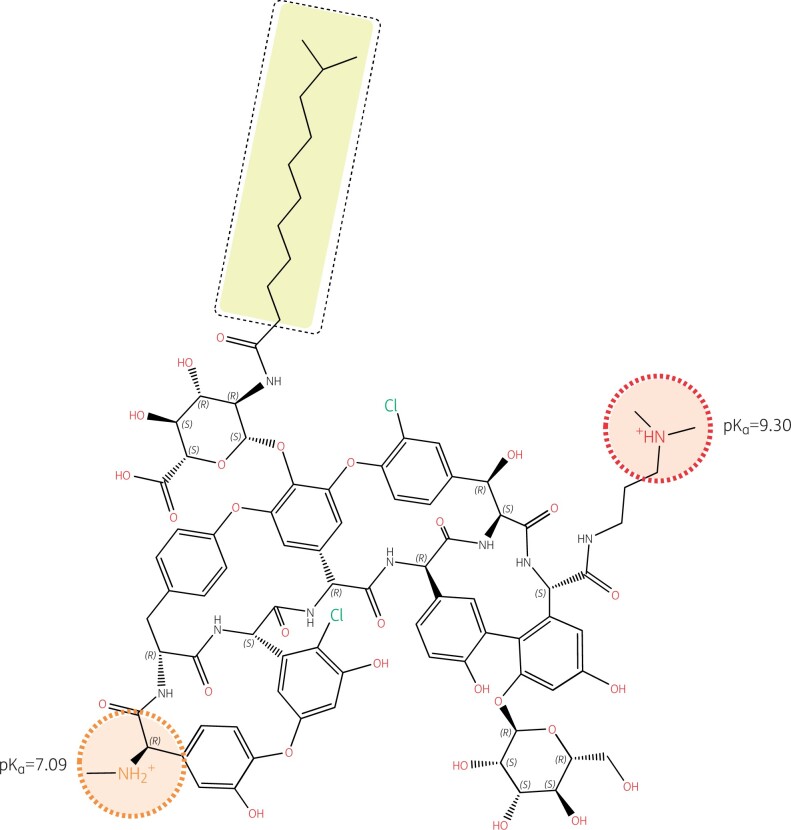
Structure of dalbavancin with emphasis on its ionizable functions and hydrophobic side chain. Like oritavancin, dalbavancin is a large molecule (projection area: 223–332 Å^2^; van der Waals volume: 1557 Å^3^) with complete lack of activity against Gram-negative pathogens and no oral bioavailability. Like vancomycin, it binds to the d-Ala-d-Ala termini of peptidoglycan precursors and, like oritavancin, its activity is also enhanced by a long hydrophobic tail (highlighted in yellow). However, the logP of dalbavancin (un-ionized form) is less positive (3.04), and its logD (ionized form) less negative (−0.25 at pH 7.4 and −1.28 at pH 5.5) than those of oritavancin, because the molecule has only two amino functions, with one having a pK_a_ of only 7.09. The molecule is thus predominantly monocationic at pH 7.4 and becomes dicationic only at acidic pH. Its weaker amphiphilic character may decrease its interaction with bacterial membranes compared with oritavancin,^94^ although enhancement of activity compared with vancomycin has been demonstrated.^95^ Note also that, in contrast to oritavancin, there is no ionizable group in close vicinity to the hydrophobic side chain. Structures and calculations were made with Marvin sketch 21.13 (Chemaxon, Budapest, Hungary).

Lipoglycopeptides were developed in response to the rise of glycopeptide-resistant *S. aureus*,^86^ and an upward trend in vancomycin MICs.^87^ Dalbavancin and oritavancin are characterized by the presence of an additional hydrophobic moiety, which is shaded yellow in Figures 4 and 5.^88–95^

These hydrophobic moieties determine their long half-lives but, most importantly, markedly improve their antimicrobial activity by increasing their membrane affinity and thus their concentration near the target.^96^ This enhancement is particularly strong for oritavancin, which is avidly taken up by macrophage and other eukaryotic cells where it accumulates mainly in lysosomes.^96^ This may partly explain its marked bactericidal effect against *S. aureus*, a phagolysosome-associated microorganism.

Dalbavancin and oritavancin both target transglycosylation, blocking elongation of peptidoglycan chains; oritavancin is more effective at inhibiting transpeptidation, which further weakens the cell wall by preventing chain crosslinking.^97^ In addition, oritavancin has a third mechanism of action that involves anchoring in the membrane through an interaction between its lipophilic chlorobiphenylmethyl moiety and bacterial lipid II, disrupting membrane integrity and conferring it with a rapid, dose-dependent bactericidal activity.^98^^,^^99^ Oritavancin’s ability to destabilize bacterial and model membranes^88^^,^^89^ further supports the concept of an antimicrobial with a ‘triple mode of action’. Because of this, and its interaction with bacterial lipid II mentioned above, oritavancin is bactericidal also against non-dividing cells, whereas dalbavancin and vancomycin are not.^91^

Oritavancin is active against some vancomycin-resistant enterococci and staphylococci,^100^ including enterococci that resist vancomycin through the VanA mechanism.^101^ Dalbavancin is not active against vancomycin-resistant enterococci carrying the VanA mechanism of resistance.^92^ This is because the 4′-chlorobiphenyl methyl moiety in oritavancin, which is responsible for the membrane interaction that causes depolarization and bacterial death, also allows oritavancin to bind more tightly than vancomycin to the d-Ala-d-Ala terminus of the cell wall peptide precursor.^93^ The enhanced binding occurs through hydrophobic interaction with the lateral methyl of d-Ala that adds to the well-known hydrogen bonds that form between the aglycon part of glycopeptides and this d-Ala-d-Ala motif. The formation of these hydrogen bonds is severely hindered through dipole–dipole repulsive effects when the terminal d-Ala is replaced by d-Lac, such as in vancomycin-resistant organisms carrying the VanA mechanism,^102^ causing vancomycin resistance. However, the binding of oritavancin is tight enough to maintain activity. Importantly, the correct positioning of the 4′-chlorobiphenyl methyl motif requires the presence of a closely apposed positive charge (Figure 4). The absence of a positive charge near the hydrophobic side chain of dalbavancin (Figure 5) explains why it shares some properties of oritavancin in relation to general membrane effects (e.g. bactericidal activity) but has no useful activity against vancomycin-resistant organisms carrying the VanA mechanism.

Dalbavancin and oritavancin are effective against a broad range of Gram-positive pathogens, including MRSA;^103^^,^^104^ however, MICs against vancomycin-resistant enterococci, even for oritavancin, remain too high for useful chemotherapeutic activity. These agents are currently approved by the FDA and EMA only for treating SSTIs. Breakpoints have been set at low values for lipoglycopeptides compared with vancomycin because of their high protein binding, which was shown to cause an increase of MICs,^105^ and to accordingly reduce their effectiveness *in vivo*.^106^^,^^107^ FDA susceptibility breakpoints are ≤0.12 mg/L for oritavancin against *S. aureus* (whether MSSA or MRSA) and *Enterococci* spp, and ≤0.25 mg/L against *Streptococcus* spp. (β-haemolytic or viridans), while those of dalbavancin are ≤0.25 mg/L for all these organisms (absence of resistant isolates precluded defining any results other than ‘Susceptible’). EUCAST MIC breakpoints for *S. aureus* (whether MSSA or MRSA) are ≤0.125 mg/L for both drugs; breakpoints are not provided for coagulase-negative staphylococci. Breakpoints for streptococci of Groups A, B, C, G, and viridans group streptococci (*Streptococcus anginosus* group only) are ≤0.25 mg/L for oritavancin and ≤0.125 mg/L for dalbavancin. For enterococci, resistance to dalbavancin is common, with both *Enterococcus faecalis and Enterococcus faecium* displaying a bimodal MIC distribution in the EUCAST database with modes at 0.06 and 16 mg/L; oritavancin has a unimodal MIC distribution for enterococci with mode at 0.08 mg/L but significant spreading to 0.25 mg/L for *E. faecium*; both drugs are classified as having insufficient evidence to determine that *Enterococcus* spp. are a good target for therapy (Table 2).

**Table 2.** Breakpoints (mg/L) of dalbavancin, oritavancin and vancomycin for Gram-positive organisms causing acute bacterial skin and skin structure infections (ABSSSI)

Pathogen	Drug^a^	FDA/CLSI M100			EUCAST	
		S	I	R	S ≤	R>
*Staphylococcus* spp.	Dalbavancin	≤0.25	^b^	^b^	0.125^c^	0.125^d^
	Oritavancin	≤0.12	^b^	^b^		
	Oritavancin, *S. aureus*				0.125^c^	0.125^d^
	Vancomycin, *S. aureus*	≤2	4–8	≥16	2	2
	Vancomycin, coagulase- negative	≤4	8–16	≥32	4	4
*Enterococcus* spp.	Dalbavancin^e^	≤0.25	^b^	^b^	IE	IE
	Oritavancin	≤0.12	^b^	^b^	IE	IE
	Vancomycin	≤4	8–16	≥32	4	4
*Streptococcus:* Groups A, B, C, and G	Dalbavancin				0.125^c^	0.125^d^
	Oritavancin				0.25^c^	0.25^d^
	Vancomycin				2	2
*Streptococcus:* β-haemolytic group	Dalbavancin^f^	≤0.25	^b^	^b^		
	Oritavancin	≤0.25	^b^	^b^		
	Vancomycin	≤1	^b^	^b^		
*Streptococcus:* viridans group	Dalbavancin, *S. anginosus* group	≤0.25	^b^	^b^	0.125^c^	0.125^d^
	Oritavancin, *S. anginosus* group	≤0.25	^b^	^b^	0.25^c^	0.25^d^
	Vancomycin	≤1	^b^	^b^	2	2

IE, there is insufficient evidence that the organism or group is a good target for therapy with the agent.

a Lipoglycopeptide MICs are method dependent (broth microdilution in the presence of 0.002% polysorbate-80 is the reference method).

b The absence of resistant clinical isolates precludes defining any results other than ‘Susceptible’.

c Isolates susceptible to vancomycin can be reported susceptible to dalbavancin and oritavancin.

d Non-susceptible isolates are rare or not yet reported. The identification and antimicrobial susceptibility test result on any such isolate must be confirmed and the isolate sent to a reference laboratory.

e For reporting against vancomycin-susceptible *E. faecalis* only.

f For reporting against *S. pyogenes, S. agalactiae*, and *S. dysgalactiae*.

## Phase III studies with lipoglycopeptides

### Dalbavancin

Dalbavancin was approved in the US in 2014,^108^ and in Europe in 2015^109^ for treating adults with ABSSSI based on the pivotal Phase III DISCOVER trials that used FDA endpoints^13^ for ABSSSI to assess non-inferiority to twice-daily vancomycin for 10–14 days with the option of switching to oral linezolid.^110^ Two identical trials were conducted in a total of 1303 patients who were randomized to dalbavancin (*n = *652) or to vancomycin with the option to switch to oral linezolid after ≥3 days (*n = *651). Early response rates at 48–72 h were similar in the dalbavancin group and the vancomycin with optional switch to linezolid group (79.7% versus 79.8%) and the proportion of patients with a ≥ 20% reduction in the infected area was also similar (88.6% versus 88.1%). Clinical success in the clinically evaluable population at day 14 (EMA endpoint) was 90.7% in the dalbavancin group versus 92.1% in the vancomycin with optional switch to linezolid group. Serious AEs occurred in 2.6% of patients receiving dalbavancin and in 4.0% of patients receiving vancomycin with optional switch to linezolid; discontinuation for an AE occurred in 2.1% of patients with dalbavancin versus 2.0% with vancomycin with optional switch to linezolid.^110^ Analysis of pooled safety data from 3000 patients in controlled trials of dalbavancin revealed AE rates of 44.9% with dalbavancin (799/1778) versus 46.8% with comparator (573/1224).^111^ Subsequently, a randomized double-blind study comparing two IV regimens of dalbavancin (1000 mg followed by a week later by 500 mg; or a single 1500 mg dalbavancin dose) established that the single dose was not inferior in terms of early response and 14 day clinical outcomes for treatment of ABSSSI.^112^

### Oritavancin

Oritavancin was approved for treating adult patients with ABSSSI^113^^,^^114^ based on the pivotal SOLO I and SOLO II studies, two studies with identical designs that demonstrated the non-inferiority of a single 1200 mg oritavancin dose to 7–10 days of twice-daily vancomycin in adults with SSTIs.^115^^,^^116^ The composite primary efficacy endpoint assessed at 48–72 h was reduction in lesion size or stop of spreading, absence of fever, and no requirement for rescue antimicrobial treatment (FDA endpoint assessed in the modified ITT population); secondary endpoints were a ≥ 20% reduction in lesion size after 48–72 h, and investigator-assessed clinical cure at day 7–14 (EMA endpoint assessed in both the modified ITT and clinically evaluable populations). Pooled efficacy analysis of results from a total of 1959 patients showed similar clinical response rates for oritavancin and vancomycin.^117^ Analysis of pooled safety data from the Phase 3 SOLO studies for 976 patients treated with oritavancin and 983 with vancomycin did not reveal significant differences in AEs (55.3 versus 56.9%) serious AEs (5.8 versus 5.9%) or treatment discontinuation for AEs (3.7 versus 4.2%). Most AEs were mild; nausea, headache and vomiting were the most frequent.^118^

A *post hoc* analysis exists of SOLO I and II patients stratified by disease severity and site of care.^119^ Most patients (70.9%) were categorized as Eron Class II (febrile, no unstable comorbidities) or class III (significant systemic toxicity, ≥1 unstable comorbidity). A study protocol variation had permitted outpatient care in US centres, with care setting determined by the investigator. Approximately 40% of patients with Class I–III infections had been treated entirely in the outpatient setting (379 with oritavancin and 388 with vancomycin). There was no difference in response rates according to disease severity or site of care. A second *post hoc* analysis showed that oritavancin was as effective as vancomycin in the outpatient setting and revealed that oritavancin had significantly better response rates for the primary endpoint among the subgroup weighing ≥100 kg, and better clinical cure rates in patients with wound infections.^120^

Retrospective assessment of real-world experience in a heterogenous cohort of 440 patients (91% with SSTIs) who received oritavancin at 26 US Centres revealed a clinical success rate of 88%.^121^ This is comparable to the combined results from the SOLO studies (93%), considering that the cohort included 39 cases of bacteraemia, osteomyelitis, synovitis, or prosthetic joint infection. Multiple oritavancin doses had been administered in 5.2% of SSTIs and 28% of other infections. MRSA was identified in 64 of 146 infections with culture results (44%).

### Economics

Several studies have assessed the potential cost savings with long-acting therapies versus inpatient treatments for SSTI.^122^^,^^123^ The cost of treatment with dalbavancin from the US perspective is approximately equivalent to the cost of 1.5 days in hospital,^124^ while a pragmatic trial comparing outcomes before (*n = *43) and after (*n = *48) introduction of an ABSSSI care pathway including dalbavancin revealed that long-acting treatment reduced LOS by 2 days.^125^ A retrospective chart review in US patients with SSTIs revealed that patients treated with oritavancin (*n = *120) had equivalent 30 day healthcare costs (12 695 versus 12 717 US$).^126^ The largest 30 day cost component with oritavancin was associated with outpatient service visits (drug acquisition and administration costs), while costs with vancomycin were largely accounted for by inpatient admissions, emergency department visits, and outpatient services.^126^ However, the study did not consider indirect costs incurred by patients receiving IV therapy and drug monitoring for 7–10 days, or benefits from reduced contact with the healthcare setting.

In addition to cost savings associated with reduced LOS and convenience of administration, the use of long-acting therapies at early discharge is also associated with favourable clinical outcomes. In the above-mentioned US retrospective chart review, oritavancin was associated with a lower 30 day subsequent hospital admission rate than vancomycin (6.1% versus 16.2%; *P = *0.003).^126^ This finding appears to be confirmed by the results of a descriptive retrospective cohort of adult inpatients with SSTIs who were discharged with single-dose oritavancin or oral step-down antibiotic therapy, revealing fewer 30 day SSTI-related readmissions in patients receiving oritavancin 7/99 (7.1%) compared with 18/100 (18.0%) with oral step-down therapy; 6 of the 7 readmissions in patients receiving oritavancin involved Gram-negative pathogens.^127^ Meanwhile, a retrospective real world study comparing single-dose dalbavancin to standard of care in age- and BMI-matched adults with ABSSSI revealed more 30 day SSTI-related readmissions in patients treated with dalbavancin 55/209 (26.32%) compared with standard of care 31/209 (14.83%; *P < *0.01).^128^

There are no clinical trials that directly compare lipoglycopeptides, however a network meta-analysis of randomized controlled trials revealed similar clinical response rates for dalbavancin, telavancin and oritavancin compared with standard of care for treatment of Gram-positive SSTIs.^129^ The associated cost analysis estimated that use of long-acting antimicrobials for MRSA in the US setting could save from 1442–6932 US$ for each cSSTI.

## Summary and future perspectives

### The value of new fluoroquinolones and long-acting lipoglycopeptides in the treatment of ABSSSI

The development of new antimicrobials is driven in large part by the urgent medical need for drugs to address multidrug-resistant pathogens; however, new agents must also address the known safety issues with the drugs already available and the high economic burden associated with ABSSSI. New agents that match these criteria are more than welcome. Dalbavancin, delafloxacin, and oritavancin meet all three of these criteria and have a place in the spectrum of antimicrobials for treating ABSSSI, where they can bridge existing gaps and overcome obstacles to treatment.

Delafloxacin and long-acting agents provide cost-effective treatment for eligible patients by allowing early discharge (delafloxacin and long-acting agents) or by avoiding admission altogether (long-acting agents). Both long-acting agents provide broad coverage for Gram-positive pathogens that includes MRSA; while delafloxacin’s coverage of both Gram-positive and Gram-negative pathogens makes it an appropriate choice for patients with ABSSSIs caused by confirmed or suspected mixed pathogens, perineal infections and abdominal surgical site infections. Long-acting agents have the added benefit of guaranteed compliance, which may provide a cost-effective alternative to hospitalization when treating infections in IV drug users when there are doubts about adherence to treatment or possible misuse of an indwelling venous access. All three of these new agents provide means to address antimicrobial stewardship goals while responding to unmet needs in ABSSSI treatment.

### Research needed to determine the future value of delafloxacin and the long-acting lipoglycopeptides dalbavancin and oritavancin

Although safety data from Phase III studies support the good tolerability of these agents (e.g. delafloxacin does not increase the risk of the QT prolongation and phototoxicity associated with other agents in its class.^72^^,^^73^), real-life data from prospective observational trials and retrospective cohort studies, as well as careful Phase IV post-marketing surveillance is necessary. Real world data may allow personalization of treatment for patients with comorbidities and clarify the roles of these agents in evolving SSTI care pathways.

## Transparency declarations

C.E. has received honoraria for lectures and advisory boards from Angelini, Astellas, AstraZeneca, Correvio, Menarini, Merck/MSD, Nabriva and Pfizer. P.M.T. has received research grants from Melinta Therapeutics for work on delafloxacin and speaker's fees from Menarini for lectures on skin and skin structure infections with focus on delafloxacin. He has received grants from Intermune for work on oritavancin, speaker's and advisor's fees from Bayer and Merck in relation to tedizolid and skin and skin structure infections; he is currently advising Correvio on the reimbursement of dalbavancin in Belgium. Richard Vernell prepared a first draft of this manuscript on behalf of Springer Healthcare Communications. This medical writing assistance was funded by A. Menarini Farmaceutica Internazionale.

This paper was published as part of a Supplement sponsored and financially supported by A. Menarini Farmaceutica Internazionale.
